# Surveillance of left ventricular function among cancer survivors

**DOI:** 10.1136/heartjnl-2025-326282

**Published:** 2025-08-05

**Authors:** Cheng Hwee Soh, Thomas H Marwick

**Affiliations:** 1 Imaging Research, Baker Heart and Diabetes Institute, Melbourne, Victoria, Australia; 2 Baker Department of Cardiometabolic Health, The University of Melbourne, Melbourne, Victoria, Australia

**Keywords:** Magnetic Resonance Imaging, Heart Failure

## Abstract

**Background:**

Cancer survivors have an increased risk of heart failure, but this is balanced by the risk of death from other causes. The results of this balance impact on the optimal time for guideline-recommended surveillance for cardiac dysfunction. This study aimed to investigate the association between cancer history and cardiac function at various times during follow-up.

**Methods:**

This cross-sectional study included participants with documented cancer history from cancer registries and matched with non-cancer controls using propensity scoring based on age, sex, diabetes and blood pressure. Cardiac function, primarily left ventricular ejection fraction (LVEF), was assessed using cardiac magnetic resonance (CMR). Multivariable binomial regression analyses were conducted to analyse the association between cancer and cardiac function.

**Results:**

Of 23 854 cancer survivors (aged 61.0±6.8 years, 60.9% female) and an equal number of matched controls, 1051 survivors and 1538 controls underwent CMR. Survivors from breast or haematological malignancies demonstrated minor differences in LVEF (59.5±6.4 vs 60.1±6.4, p<0.001) and global circumferential strain (−22.4±3.5 vs −22.6±3.5, p<0.001) compared with controls. Analysis stratified by time since cancer diagnosis revealed that both LVEF (p=0.014) and global circumferential strain (p=0.045) were less likely to be impaired with increasing time from diagnosis. Cancer survivors (prevalence ratio (PR)=1.19 (95% CI 1.05 to 1.35), p=0.006), particularly breast cancer (PR=1.39 (95% CI 1.18 to 1.64), p<0.001), were associated with low LVEF (≤55%) after adjusting for age, sex, years since cancer diagnoses and blood pressure medication.

**Conclusions:**

Compared with people without cancer, cancer survivors have a higher risk of subclinical cardiac dysfunction. However, dysfunction is less common with increasing time since cancer diagnosis. These findings suggest prioritising cardiac monitoring early in survivorship, especially in breast cancer survivors.

**WHAT IS ALREADY KNOWN ON THIS TOPIC**

- Cancer survivors are at increased risk of long-term cardiovascular complications, including heart failure, due to both treatment-related cardiotoxicity (eg, anthracyclines, chest radiotherapy) and cumulative exposure to conventional cardiovascular risk factors.

**WHAT THIS STUDY ADDS**

- Cancer survivors, particularly those with a breast cancer history, demonstrate significantly higher risk of subclinical cardiac dysfunction.
- The risk difference in subclinical cardiac dysfunction between cancer survivors and the non-cancer cohort diminishes at longer follow-up periods since cancer diagnosis.

**HOW THIS STUDY MIGHT AFFECT RESEARCH, PRACTICE OR POLICY**

- This study emphasises the importance of implementing systematic cardiac monitoring protocols early in the survivorship journey to enable timely intervention and prevent future cardiovascular complications.
- Future research should investigate the long-term trajectories of cardiac function across different cancer types while accounting for treatment modalities, enabling the development of risk-stratified monitoring protocols to optimise cardiovascular care in cancer survivors.

## Introduction

 Cancer survivors are at risk of cardiovascular (CV) complications with higher rates of CV morbidity compared with non-cancer populations.^1^ Treatment-related cardiac injury potentially extends beyond initial treatment periods,^2^ because this is attributable to the cardiotoxic effects of chemotherapy and radiotherapy,^3^ and lifetime exposure to heart failure (HF) risk factors.^4^ Consequently, HF is an ongoing concern in many survivors, and especially those with cancers which frequently involve anthracycline and/or chest radiotherapy. Current guidelines support the use of echocardiography every 5 years in survivors at moderate risk of HF,^1 5^ although assessment of risk is difficult in primary care because of limited details of chemotherapy and radiotherapy. Left ventricular ejection fraction (LVEF) remains the most widely used parameter for assessment of left ventricular (LV) function^6^; using cardiac magnetic resonance (CMR), a normal LVEF ranges from 51% to 76% in men and from 52% to 79% in women,^7^ but values <55% are broadly accepted to indicate potential cardiac dysfunction.^8^ While extensive research has explored short-term cardiotoxic effects, significant gaps remain in understanding the long-term cardiac consequences for cancer survivors. This study sought to compare CMR parameters between cancer survivors at various follow-up durations since diagnosis and non-cancer controls.

## Methods

### Study design and setting

This cross-sectional analysis uses the UK Biobank database, a large, population-based, prospective cohort study with nearly 500 000 participants aged 35 years or older recruited from 2006 to 2010.^9^ The baseline assessment obtained sociodemographic, health and lifestyle information, physical assessments (blood pressure, arterial stiffness measurement, body composition analysis, hand grip strength testing, bone densitometry, spirometry, fitness test with electrocardiography) and biological sample collection (blood, urine and saliva). A subgroup of these patients underwent CMR imaging. The UK Biobank imaging study was launched in 2015, with an aim of scanning 20% of the original cohort.^10^ Version 3.4 of the UK Biobank database was used for this study.

### Ethical approval

The study followed the guidelines outlined in the Declaration of Helsinki, and written informed consent was provided by all participants.

### Cancer diagnosis

Participants were stratified into cancer survivors and non-cancer controls using the International Classification of Diseases-10th Revision (ICD-10) codes for cancer diagnoses (field ID 40006). Cancer types were identified using codes C00–C97, excluding melanoma and other skin malignant neoplasms (C43–C44). Diagnosis dates (field ID 40005) were recorded during ICD-10 documentation. As this was a cross-sectional baseline analysis, only participants with cancer diagnoses prior to their consent date (field ID 200) were included in the cohort of cancer survivors.

### Cardiac magnetic resonance

Cardiac imaging phenotypes of four cardiac chambers (left ventricle (LV), right ventricle, left atrium, right atrium), along with regional phenotypes (including LV myocardial wall thickness and strain), were derived using an automated machine learning pipeline trained on 3975 participant images.^11^ The analysis calculated comprehensive cardiac parameters including end-diastolic and end-systolic volumes, stroke volume, ejection fraction (EF) and myocardial mass. Motion tracking was performed using non-rigid image registration to assess myocardial displacement and strain.^12^ Long-axis image analysis focused on segmenting left and right atria using neural networks, determining atrial areas and longitudinal diameters from four-chamber and two-chamber views.^13^ Atrial volumes were calculated using a biplane area-length formula, with motion tracking performed to evaluate longitudinal strain.^14^

### Statistical analysis

Baseline characteristics were reported using descriptive statistics, with continuous variables presented as mean±SD and categorical variables as count (percentage). Student’s t-tests and χ^2^ tests compared differences between cancer survivors and non-cancer controls. Due to substantial baseline differences in CV risk factors, propensity score matching was performed to create a comparable control cohort. A propensity score was estimated using logistic regression, with age, sex, diabetes status and use of blood pressure medications as covariates. Individuals were then matched in a 1:1 ratio using exact matching on all four variables. Following matching, the matched dataset was used for outcome comparisons. Although exact matching was performed, the matching variables were also included as covariates in multivariable regression models to further adjust for any residual imbalance.

CMR-assessed participants with and without a cancer history were compared. Cardiac parameters, including the primary outcome in LVEF, were analysed as continuous variables using Student’s t-tests. Categorical outcomes, including LVEF ≤55%, left atrial volume index (LAVi >34 mL/m^2^) and global longitudinal strain (GLS ≥−16%), were compared using χ^2^ tests. Sensitivity analyses included stratifying survivors by cancer type (breast/haematological malignancies vs other cancer types) and duration since diagnosis (≤1, 1–5, 5–10, ≥10 years), as well as lowering LVEF cut-off values to ≤50%. Breast cancer was defined as malignant neoplasms of breast, while haematological malignancies were defined as malignant neoplasms, stated or presumed to be primary, of lymphoid, haematopoietic and related tissue.

Multivariable binomial regression models, reported as prevalence ratio (PR) and 95% CI, and linear regression models, reported as β and SE, were conducted to analyse the associations between cancer diagnosis and cardiac parameters, adjusting for covariates including age, sex, years since diagnosis, blood pressure medication, diabetes, cholesterol-lowering medication and ischaemic heart disease. Histograms were used to assess the normality of the outcome variables, as well as the residuals from linear regression models. Statistical significance was set at p<0.05, with all analyses conducted using R (R Foundation for Statistical Computing).

## Results

### Participant characteristics

Of the total 487 031 participants, 23 854 were identified as having a history of cancer diagnoses prior to consenting to the UK Biobank study. The three most common cancer types were malignant neoplasms of breast (37.1%), male genital organs (19.1%) and digestive organs (16.5%) (online supplemental table 1). Compared with cancer survivors (mean age 61.0 years, 60.9% females), non-cancer participants (n=463 177) were significantly younger (mean age 56.8 years) and had fewer comorbidities. Table 1 demonstrates the characteristics of the cancer survivors and non-cancer controls matched based on age, sex, diabetes and blood pressure medication. After propensity matching, the number of females progressively increased as the number of years since cancer diagnosis increased (≤1 year: 52.3%; 1–5 years: 55.7%; 5–10 years: 62.7%; ≥10 years: 71.4%). No significant difference was identified in the prevalence of comorbidities between cancer survivors and controls.

**Table 1:** Baseline characteristics of participants in the matched cohort of participants with and without cancer history

	Control (n=1538)	Cancer dx ≤1 year (n=151)	Cancer dx 1–5 years (n=370)	Cancer dx 5–10 years (n=271)	Cancer dx ≥10 years (n=259)	Overall P value
Age, years	59.1±6.8	59.0±6.8	58.7±7.0	59.4±6.9	58.9±6.4	0.75
Female, n (%)	892 (58.0)	79 (52.3)	206 (55.7)	170 (62.7)	185 (71.4)	**<0.001**
Systolic blood pressure, mm Hg	140.0±19.5	140.5±19.3	137.4±18.7	139.6±20.2	137.2±19.4	0.074
Body mass index, kg/m^2^	26.7±4.2	26.9±4.7	26.8±4.4	26.4±3.9	26.7±4.5	0.76
Diabetes, n (%)	100 (6.5)	9 (6.0)	24 (6.5)	19 (7.0)	23 (8.9)	0.69
Ischaemic heart disease, n (%)	145 (9.4)	25 (16.6)	33 (8.9)	23 (8.5)	26 (10.0)	0.062
Chronic renal failure, n (%)	49 (3.2)	9 (6.0)	18 (4.9)	12 (4.4)	10 (3.9)	0.29
Blood pressure medication, n (%)	164 (10.7)	20 (13.2)	45 (12.2)	27 (10.0)	22 (8.5)	0.51
Cholesterol-lowering meds, n (%)	158 (10.3)	17 (11.3)	40 (10.8)	28 (10.3)	20 (7.7)	0.72
International Physical Activity Questionnaire, n (%)						
Low	236 (15.3)	32 (21.2)	65 (17.6)	42 (15.5)	28 (10.8)	0.26
Moderate	544 (35.4)	50 (33.1)	128 (34.6)	95 (35.1)	90 (34.7)	
High	499 (32.4)	42 (27.8)	112 (30.3)	96 (35.4)	88 (34.0)	

Propensity matching was performed based on age, sex, diabetes and blood pressure medication. However, the difference in the proportion of male and female between cancer survivors at each subgroup and controls remained signficant.

dx, diagnosis.

### CMR measurements in all survivors

Within the matched cohort of survivors and controls, 1051 and 1538 cancer survivors and controls, respectively, completed their baseline CMR assessment (online supplemental table 2). For the primary outcome, survivors had slightly lower LVEF (59.2±6.5%) compared with controls (60.1±6.4%, p<0.001). The differences between survivors and controls’ LV global circumferential strain (GCS) were also statistically significant (−22.2±3.5% vs −22.6±3.5%, p=0.001), whereas the differences in the cohorts’ GLS were insignificant (p=0.874).

The cohort of survivors was then further stratified based on years since cancer diagnosis. Table 2 shows that survivors within a year since cancer diagnosis (n=151) had the worst cardiac function, particularly LVEF (58.7±6.7%), LV end-systolic volume (61.7±21.0 mL), LV end-diastolic volume (147.6±34.9 mL) and GCS (−21.8±3.6%). On the other hand, 259 survivors who were 10 years or more after cancer diagnosis showed a similar cardiac function as controls (LVEF: 59.8±6.3%; LV cardiac output: 5.3±1.2 L/min; GCS: −22.5±3.5; and maximum LAVi: 38.8±10.5 mL/m^2^). There was no difference in LV cardiac output (p=0.777), as well as GLS (p=0.576) and left ventricular mass index (LVMi) (p=0.192). Categorising the main CMR parameters, the prevalence of LVEF ≤55% was present in 18.9% of controls, compared with 25.2% (p=0.078) among those studied at ≤1 year after diagnosis, 24.3% (p=0.022) at 1–5 years, 26.6% (p=0.005) at 5–10 years and 20.8% (p=0.009) at ≥10 years.

**Table 2:** Comparison of participants’ cardiac function, stratified by years since cancer diagnoses

	Control (n=1538)	Cancer dx ≤1 year (n=151)		Cancer dx 1–5 years (n=370)		Cancer dx 5–10 years (n=271)		Cancer dx ≥10 years (n=259)		Overall P value
		Value	P value*	Value	P value*	Value	P value*	Value	P value*	
LA measurements										
LA enlargement (LAVi >34) (%)	47 (3.1)	7 (4.6)	0.42	6 (1.6)	0.18	9 (3.3)	0.97	3 (1.2)	0.13	0.14
LA minimum volume index (mL/m^2^)	16.1±8.7	16.7±8.7	0.94	15.7±8.9	0.93	16.2±9.1	0.99	15.6±6.3	0.87	0.66
LA maximum volume index (mL/m^2^)	38.9±12.1	39.2±11.3	0.99	38.4±12.6	0.95	39.2±11.9	0.99	38.8±10.5	0.99	0.93
LA ejection fraction (%)	60.3±10.3	59.1±10.4	0.60	60.7±9.6	0.96	60.3±9.5	0.99	60.9±8.0	0.91	0.45
LV measurements										
LV cardiac output (L/min)	5.3±1.2	5.4±1.2	0.45	5.4±1.3	0.79	5.2±1.1	0.87	5.3±1.2	0.99	0.23
LV stroke volume (mL)	84.9±18.1	86.0±19.4	0.95	84.8±18.5	0.99	83.5±17.5	0.79	82.7±17.3	0.38	0.27
LV end-systolic volume (mL)	57.4±18.6	61.7±21.0	0.055	59.7±19.3	0.20	58.7±17.6	0.82	56.6±18.0	0.97	**0.014**
LV end-diastolic volume (mL)	142.2±31.8	147.6±34.9	0.26	144.5±32.4	0.73	142.2±29.8	0.99	139.2±30.0	0.63	0.081
LV mean myocardial wall thickness (mm)	5.7±0.8	5.7±0.7	0.71	5.6±0.7	0.92	5.6±0.8	0.99	5.5±0.7	**0.048**	**0.035**
LV mass index (g/m^2^)	44.9±8.2	45.8±8.2	0.67	44.9±8.3	0.99	45.1±7.7	0.99	43.7±8.5	0.21	0.12
LV global circumferential strain (%)	−22.6±3.5	−21.8±3.6	**0.042**	−22.0±3.5	**0.019**	−22.3±3.5	0.60	−22.5±3.5	0.95	**0.004**
LV GLS (%)	−18.6±2.8	−18.3±3.0	0.70	−18.5±2.9	0.97	−18.8±2.9	0.90	−18.7±2.7	0.99	0.49
LV global radial strain (%)	46.2±8.5	44.1±8.5	**0.028**	44.9±9.1	0.059	45.7±8.5	0.88	46.2±9.0	0.99	**0.006**
Abnormal GLS (≥ −16%) (%)	259 (16.8)	29 (19.2)	0.53	69 (18.6)	0.45	41 (15.1)	0.54	40 (15.4)	0.64	0.67
LV mass index (g/m^2^)	44.9±8.2	45.8±8.2	0.67	44.9±8.3	0.99	45.1±7.7	0.99	43.7±8.5	0.21	0.12
LV ejection fraction (%)	60.1±6.4	58.7±6.7	0.064	59.1±6.5	**0.044**	59.1±6.5	0.11	59.8±6.3	0.93	**0.003**
LV systolic dysfunction (EF ≤55) (%)	290 (18.9)	38 (25.2)	0.078	90 (24.3)	**0.022**	72 (26.6)	**0.005**	54 (20.8)	0.50	**0.009**

* P values indicate differences between subgroups of survivors stratified based on cancer diagnosis and control.

dx, diagnosis; EF, ejection fraction; GLS, global longitudinal strain; LA, left atrium; LAVi, left atrial volume index; LV, left ventricle.;

### CMR measurements in ‘at risk’ survivors

Stratifying the survivors into subpopulations of cardiotoxicity risk (breast/haematological malignancies vs other cancer types) showed that most findings were consistently different from controls. However, although LV dimensions were less among the survivors who were at higher risk (perhaps reflecting prevalence in women), LV function markers seemed worse in the ‘other cancers’ group (table 3).

**Table 3:** Comparison of participants’ cardiac structure and function, stratified by cancer type

	Control (n=1538)	Breast/haematological malignancies (n=534)		Other cancer (n=517)		Overall P value
		Value	P value*	Value	P value*	
Age, years	59.1±6.8	58.1±6.8	**0.006**	59.8±6.7	0.10	**<0.001**
Female (%)	892 (58.0)	467 (87.5)	**<0.001**	173 (33.5)	**<0.001**	**<0.001**
Body surface area (m^2^)	1.8±0.2	1.8±0.2	**<0.001**	1.9±0.2	**<0.001**	**<0.001**
LA measurements						
LA ejection fraction (%)	60.3±10.3	60.5±9.2	0.95	60.4±9.4	0.99	0.95
LA minimum volume index (mL/m^2^)	16.1±8.7	16.1±8.4	0.99	15.9±8.4	0.80	0.81
LA maximum volume index (mL/m^2^)	38.9±12.1	39.1±11.7	0.96	38.5±11.8	0.79	0.73
LA enlargement (max LAVi >34) (%)	47 (3.1)	14 (2.6)	0.34	11 (2.1)	0.72	0.52
LV measurements						
LV ejection fraction (%)	60.1±6.4	59.5±6.4	0.12	58.9±6.5	**0.001**	**<0.001**
LV global circumferential strain (%)	−22.6±3.5	−22.4±3.5	0.57	−21.9±3.5	**<0.001**	**<0.001**
LV GLS (%)	−18.6±2.8	−18.8±2.9	0.36	−18.4±2.8	0.23	**0.047**
LV global radial strain (%)	46.2±8.5	46.1±8.8	0.92	44.5±8.9	**<0.001**	**<0.001**
Abnormal GLS (≥−16%) (%)	259 (16.8)	89 (16.7)	0.98	90 (17.4)	0.82	0.94
LV cardiac output (L/min)	5.3±1.2	5.1±1.1	**0.004**	5.5±1.2	**<0.001**	**<0.001**
LV stroke volume (mL)	84.9±18.1	79.4±16.0	**<0.001**	89.0±18.8	**<0.001**	**<0.001**
LV end-systolic volume (mL)	57.4±18.6	54.9±17.6	**0.025**	63.1±19.2	**<0.001**	**<0.001**
LV end-diastolic volume (mL)	142.2±31.8	134.3±28.7	**<0.001**	152.1±32.0	**<0.001**	**<0.001**
LV mean myocardial wall thickness (mm)	5.7±0.8	5.3±0.6	**<0.001**	5.9±0.7	**<0.001**	**<0.001**
LV mass index (g/m^2^)	44.9±8.2	42.3±7.4	**<0.001**	47.4±8.2	**<0.001**	**<0.001**
LV systolic dysfunction (EF ≤55) (%)	290 (18.9)	120 (22.5)	0.081	134 (25.9)	**<0.001**	**0.002**

Haematological malignancies were defined as malignant neoplasms, stated or presumed to be primary, of lymphoid, haematopoietic and related tissue.

* P values indicate differences between cancer types and control.

**Figure 1:**
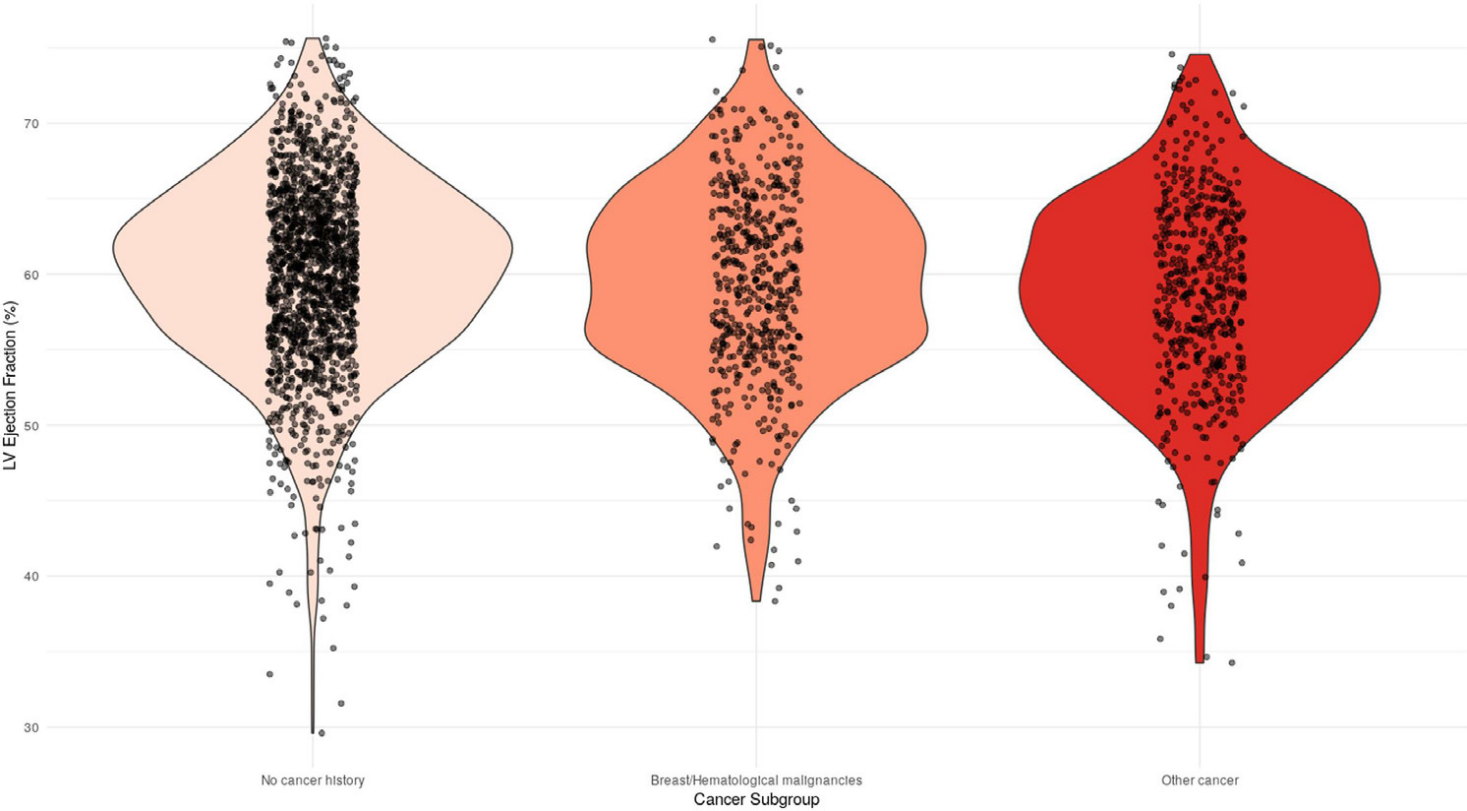
Distribution of LV ejection fraction according to presence and type of cancer history. Breast cancer was defined as malignant neoplasms of breast. Haematological malignancies were defined as malignant neoplasms, stated or presumed to be primary, of lymphoid, haematopoietic and related tissue. LV, left ventricular.

**Figure 2:**
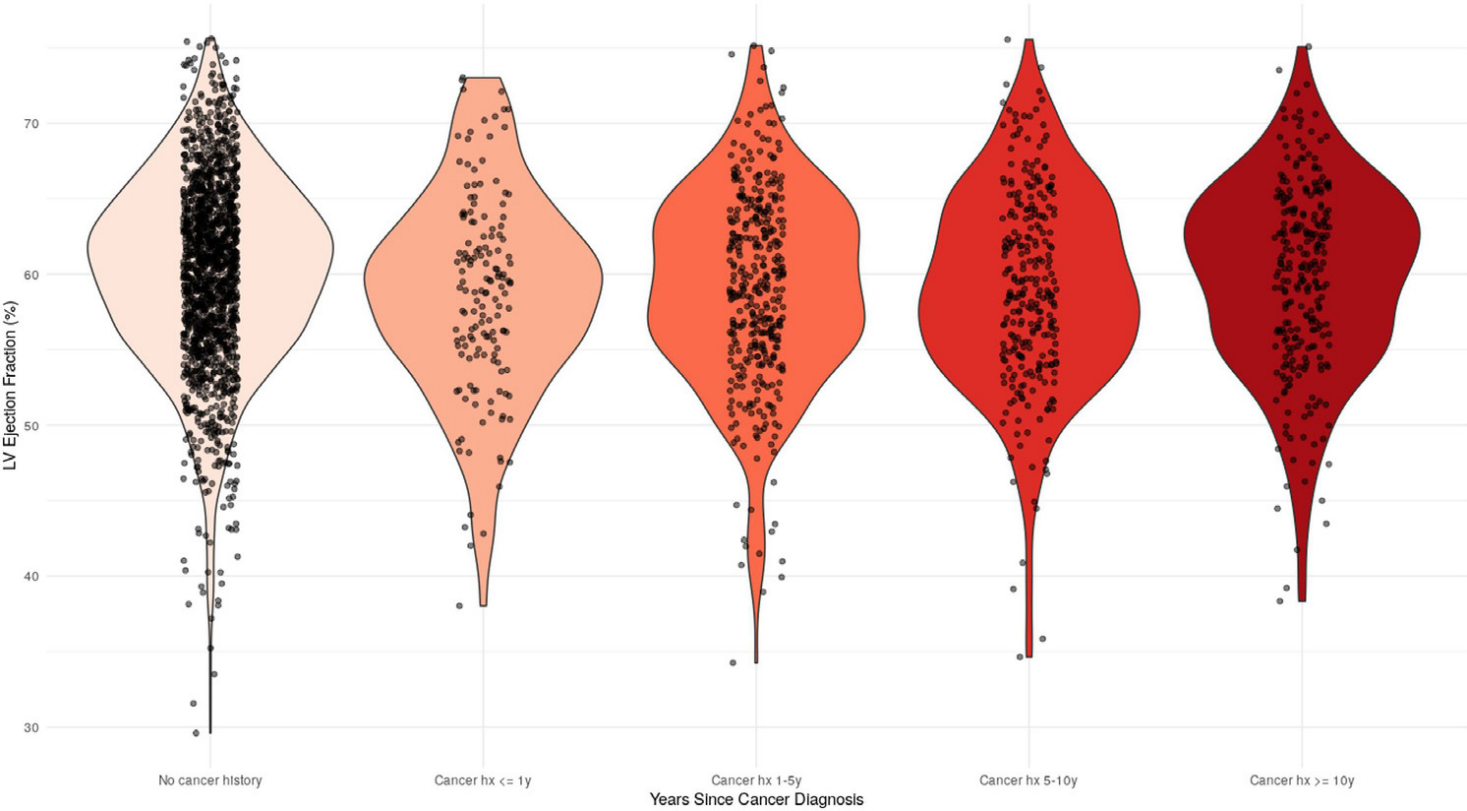
Distribution of LV ejection fraction according to cancer history, stratified via years since cancer diagnosis. hx, history; LV, left ventricular.

EF, ejection fraction; GLS, global longitudinal strain; LA, left atrium; LAVi, left atrial volume index; LV, left ventricle.;

Based on these stratifications, violin plots were generated to visualise the distribution of survivors and controls’ LVEF. Figure 1 showed that despite statistically significant differences in LVEF, a similar distribution was identified in the three cohorts. On the other hand, figure 2 demonstrates a progressive increment in LVEF from ≤1, 1–5, 5–10 to ≥10 years since cancer diagnosis.

### Associations of LV dysfunction

Comparing the characteristics of participants with low LVEF (≤55%) to those with normal LVEF, online supplemental table 3 shows that there were significantly fewer females (37.3% vs 60.6%), more cancer survivors (47.9% vs 41.1%) and higher prescription rates for blood pressure (14.9% vs 9.6%) and cholesterol-lowering medications (17.3% vs 8.3%).

Multivariable binomial regression was conducted to identify the association between the history of cancer diagnoses and LVEF (table 4). After adjusting for age, sex, blood pressure medication and years since cancer diagnosis, cancer was shown to be significantly associated with low LVEF (PR 1.19 (95% CI 1.05 to 1.35), p=0.006). Further stratifying the cancer diagnoses to subpopulations of cancer types, breast cancer was shown to be significantly associated with low LVEF (OR 1.39 (95% CI 1.18 to 1.64), p<0.001), whereas the association between blood-related cancer and low LVEF was borderline significant (PR 1.18 (95% CI 0.99 to 1.39), p=0.055). Additional covariates, including diabetes, cholesterol-lowering medication and ischaemic heart disease, were then added into the regression model, and breast cancer remained significantly associated with low LVEF (PR 1.38 (95% CI 1.17 to 1.62), p<0.001). A sensitivity analysis defining low LVEF as ≤50% was performed, and the multivariable binomial regression showed stronger association between breast cancer and low LVEF after adjusting for all covariates (PR 2.01 (95% CI 1.41 to 2.83), p<0.001) (online supplemental table 4).

**Table 4:** Independent associations of LV systolic dysfunction (LVEF ≤55%)

	Clinical variables		Cancer variables		All variables	
	PR (95% CI)	P value	PR (95% CI)	P value	PR (95% CI)	P value
Age at consent	0.99 (0.99 to 1.00)	**0.021**	0.99 (0.99 to 0.99)	**0.029**	0.99 (0.99 to 1.00)	**0.003**
Female	0.43 (0.39 to 0.47)	**<0.001**	0.39 (0.34 to 0.44)	**<0.001**	0.40 (0.35 to 0.45)	**<0.001**
Cancer history at baseline	1.19 (1.05 to 1.35)	**0.006**	–	–	–	–
Breast cancer	–	–	1.39 (1.18 to 1.64)	**<0.001**	1.38 (1.17 to 1.62)	**<0.001**
Haematological malignancies	–	–	1.18 (0.99 to 1.39)	0.055	1.17 (0.98 to 1.37)	0.072
Other cancer	–	–	1.04 (0.88 to 1.21)	0.63	1.04 (0.88 to 1.21)	0.65
Years since cancer diagnosis	1.00 (0.99 to 1.01)	0.89	1.00 (0.99 to 1.00)	0.19	1.00 (0.99 to 1.00)	0.17
Blood pressure medication	0.95 (0.84 to 1.07)	0.39	0.95 (0.84 to 1.07)	0.42	0.86 (0.75 to 0.98)	**0.029**
Diabetes	–	–	–	–	1.08 (0.92 to 1.25)	0.32
Cholesterol-lowering medication	–	–	–	–	1.07 (0.94 to 1.22)	0.33
Ischaemic heart disease	–	–	–	–	1.41 (1.25 to 1.57)	**<0.001**

Cancer history, particularly breast cancer, was shown to be significantly associated with LV systolic dysfunction after adjusting for age, sex, blood pressure medication, years since cancer diagnosis, diabetes, cholesterol-lowering medication and ischaemic heart disease.

LV, left ventricular; LVEF, left ventricle ejection fraction; PR, prevalence ratio.

Online supplemental table 5 shows the associations between cancer diagnosis and key cardiac findings (LVEF, LV myocardial mass, GLS and GCS), adjusted for age, sex, blood pressure medication and years since diagnosis. Cancer diagnoses were shown to be associated with lower LVEF (β: −0.5 (SE 0.2), p=0.027) and pathological change in GCS (β: 0.2 (SE 0.1), p=0.049). No major deviation from normality was identified for all four outcome variables, as well as the residuals from the corresponding linear regression models (online supplemental figures 1 and 2). On the other hand, cancer was not associated with categorical outcomes for abnormal GLS (≥−16%; p=0.73 or LAVi >34 mL/m^2^; p=0.42, respectively) after adjustment for covariates (online supplemental table 6).

## Discussions

In this study of cardiac function in cancer survivors using CMR, with most survivors (35%) being 1–5 years after diagnosis, there were significant differences in LVEF, GCS and LV end-systolic volume between survivors and controls. Notably, cardiac function was less likely to be impaired with increasing time since cancer diagnosis. Cancer diagnoses, particularly breast cancer, were independently associated with low LVEF after adjusting for key covariates (central illustration, online supplemental file 1).

This study highlights cancer survivors’ increased risk of subclinical cardiac dysfunction, primarily manifested through reduced LVEF. Transient LVEF decreases can occur following cancer treatments like anthracyclines or trastuzumab.^15^ While initial recovery might suggest reversibility, this may create a ‘fertile field’ for future myocardial injury.^16^ Supporting these concerns, broader epidemiological evidence has shown that adult cancer survivors face significantly higher risks of hospitalisation for various somatic diseases compared with cancer-free controls.^17^ Despite this study’s cross-sectional design, the findings underscore the need for longitudinal research to track LVEF trajectories and better understand survivors’ long-term CV health.

Myocardial strain patterns provide valuable insights into cardiac disease progression, with longitudinal, circumferential and radial strain exhibiting distinct directional changes that may predict future cardiovascular disease (CVD) risk.^18 19^ Research has shown that in patients with hypertension with LV hypertrophy, circumferential strain increases while longitudinal strain and radial strain decrease.^20^ This differential pattern of strain changes may serve as an early marker of cardiac dysfunction, often preceding detectable changes in traditional measures like EF. Although our study found no significant difference in LVMi between survivors and controls, the observed elevation in GCS among survivors is noteworthy. This finding suggests a compensatory mechanism where increased circumferential function may be masking early cardiac dysfunction. The heightened GCS may therefore indicate an increased risk for subsequent systolic dysfunction, potentially identifying a subset of survivors who could benefit from closer CV monitoring despite normal conventional cardiac parameters. Interestingly, while a small but statistically significant reduction in LVEF was observed among cancer survivors compared with controls, no corresponding difference was identified in CMR-derived GLS. This appears counterintuitive, as GLS is generally considered a more sensitive marker of subclinical LV dysfunction and is known to decline earlier than LVEF in higher risk populations. However, the evidence for the superiority of GLS derives from echocardiography and is primarily driven by the inferior performance of echo-EF. The same cannot be expected with CMR measurements, where feature-tracking GLS is obtained at lower frame rate than with echocardiography.

The temporal analysis further supports these findings, with compromised cardiac function being most pronounced within the first year after cancer diagnosis. Previous research documented CV events primarily occurring within the first year after diagnosis, with a secondary risk peak between the fourth and fifth years.^21^ Our study revealed a notable pattern in LVEF impairment across different survivor subgroups: the prevalence of low LVEF was high among survivors within their first year of cancer diagnosis, followed by a lower prevalence in the subgroup aged 1–5 years after diagnosis, and then a subsequent increase among those 5–10 years after diagnosis. This biphasic distribution pattern suggests the need for sustained CV monitoring and consideration of early cardioprotective interventions, regardless of the time since cancer diagnosis. While our cross-sectional analysis cannot determine individual trajectories of cardiac function, these findings highlight potential periods of increased CV vulnerability that warrant further investigation through longitudinal studies.

Population-based studies have demonstrated that survivors across various cancer types face elevated risks of CVD compared with the general population, with notable variations between cancer sites.^22^ In breast cancer, cardiotoxicity was notably increased with concurrent trastuzumab and anthracyclines, with LV dysfunction rates in 20.1% after 5 years.^23^ Historically, left-sided breast cancer radiotherapy in the early 1980s was associated with higher CV-related mortality due to larger irradiation fields, though the risk of ischaemic heart disease from breast cancer radiation has substantially decreased over time.^23^ On the other hand, the treatment of haematological malignancies employs diverse therapeutic approaches, including conventional chemotherapy (anthracyclines, alkylating agents, antimetabolites), targeted therapies (tyrosine kinase inhibitors, monoclonal antibodies) and emerging immunotherapies (checkpoint inhibitors, chimeric antigen receptor T-cell therapy, stem cell transplantation), each with distinct cardiotoxic profiles that warrant careful consideration in treatment planning.^24^ These site and treatment-specific variations in cardiotoxicity profiles underscore the importance of tailored CV monitoring strategies for different cancer populations.

### Limitations

This study has several important limitations. First, the UK Biobank cohort is known for ‘healthy volunteer’ selection bias, with participants typically being healthier, better educated and having fewer self-reported health conditions compared with the general population. Notably, all-cause mortality and cancer incidence were lower than expected for the age range studied.^25^ Second, CMR data were available for <10% of participants and even lower in cancer survivors, with only 4.4% completion rate. This limited sampling may underestimate the true impact of cancer history on cardiac function, as participants with worsening cardiac function might have been less likely to undergo CMR. Third, the study lacked detailed cancer treatment history, a critical limitation since treatments like chest radiotherapy and chemotherapy can cause cardiotoxic effects and therapy-related cardiac dysfunction.^26^ The absence of this information may have introduced residual confounding, particularly in analyses stratified by time since cancer diagnosis, where treatment exposures likely differ across groups. As a result, while these stratified findings provide preliminary insights, they should be interpreted with caution and do not directly inform current cardiac surveillance protocols without better adjustment for treatment-related risk. Lastly, to date, most participants in the UK Biobank study (~98%) did not undergo repeat CMR imaging, limiting our ability to assess longitudinal changes in cardiac structure or function. Future research should comprehensively document treatment histories and examine long-term trajectories of survivors’ cardiac function to develop more precise screening guidelines for subclinical cardiac dysfunction and potentially reduce future CVD incidence.

## Conclusions

This study provides empirical evidence of subclinical cardiac dysfunction among cancer survivors compared with non-cancer controls. The duration since cancer diagnosis emerged as a critical factor, with survivors in the first year after diagnosis demonstrating the most compromised cardiac function. These findings underscore the importance of targeted cardiac screening within the first year of cancer diagnosis despite asymptomatic presentation to detect and address subclinical cardiac dysfunction early, potentially enabling timely cardioprotective interventions.

## Supplementary material

10.1136/heartjnl-2025-326282 online supplemental file 1

10.1136/heartjnl-2025-326282 online supplemental file 2

## Data Availability

Data are available upon reasonable request.
