# Supplementary figures and images for: Surveillance of left ventricular function among cancer survivors
Source: Heart. 2025 Aug 5;111(24):e326282. doi: 10.1136/heartjnl-2025-326282 (PMC12703235; doi:10.1136/heartjnl-2025-326282)

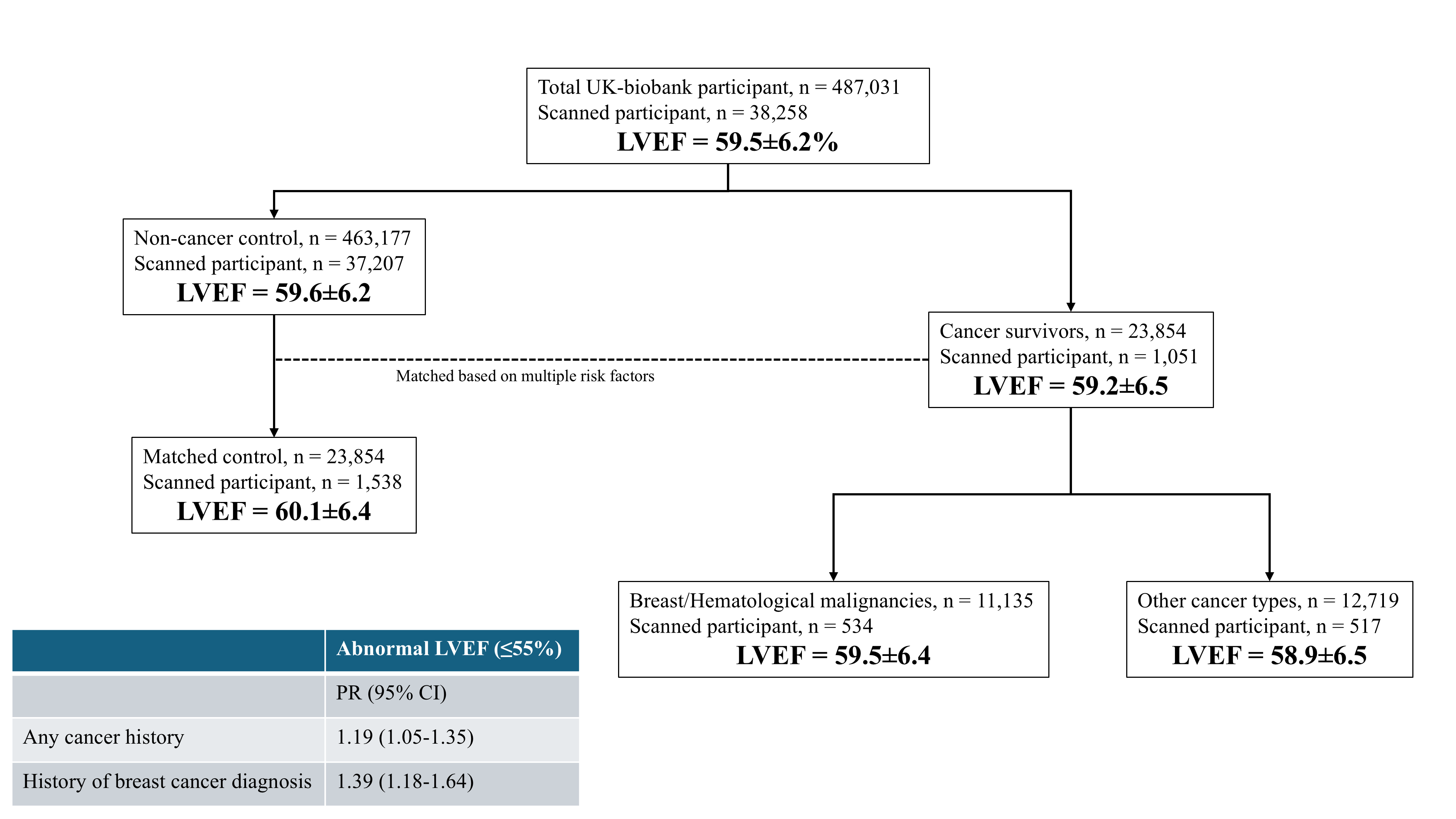

Supplement: online supplemental file 1 [file heartjnl-111-24-s001.tif]
