# Supplementary material for: Surveillance of left ventricular function among cancer survivors
Source: Heart. 2025 Aug 5;111(24):e326282. doi: 10.1136/heartjnl-2025-326282 (PMC12703235; doi:10.1136/heartjnl-2025-326282)

Supplementary Table 1: Baseline characteristics of participants with and without cancer history.

|  | Control (n=463,177) |  | Cancer at baseline (n=23,854) |  | p-value |
| --- | --- | --- | --- | --- | --- |
| Age, years | 56.8 ± 8.1 |  | 61.0 ± 6.8 |  | **<0.001** |
| Female (%) | 249,537 (53.9) |  | 14,517 (60.9) |  | **<0.001** |
| Systolic blood pressure, mmHg | 140 ± 20 |  | 142 ± 20 |  | **<0.001** |
| Body mass index, kg/m2 | 27.4 ± 4.8 |  | 27.5 ± 4.9 |  | **0.001** |
| Diabetes (%) | 42,283 (9.1) |  | 2,841 (11.9) |  | **<0.001** |
| Ischemic heart disease (%) | 55,955 (12.1) |  | 3,711 (15.6) |  | **<0.001** |
| Chronic renal failure (%) | 21,903 (4.7) |  | 2,114 (8.9) |  | **<0.001** |
| Blood pressure medication (%) | 51,780 (11.2) |  | 3,193 (13.4) |  | **<0.001** |
| Cholesterol lowering medication (%) | 48,416 (10.5) |  | 2,796 (11.7) |  | **<0.001** |
| IPAQ (%) |  |  |  |  | **<0.001** |
| Low | 66,206 (14.3) |  | 3,568 (15.0) |  |  |
| Moderate | 145,372 (31.4) |  | 7,552 (31.7) |  |  |
| High | 146,932 (31.7) |  | 6,800 (28.5) |  |  |
| Years since cancer diagnosis, years | - |  | 7.4 ± 6.7 |  |  |
| ≤ 1 year (%) |  |  | 3,461 (14.5) |  |  |
| 1-5 years (%) |  |  | 8,388 (35.2) |  |  |
| 5-10 years (%) |  |  | 6,371 (26.7) |  |  |
| ≥ 10 years (%) |  |  | 5,634 (23.6) |  |  |
| Site of cancer |  |  |  |  |  |
| Breast | - |  | 8,854 (37.1%) |  |  |
| Male genital organs | - |  | 4,567 (19.1%) |  |  |
| Digestive organs | - |  | 3,947 (16.5%) |  |  |
| Hematopoietic, lymphoid | - |  | 2,418 (10.1%) |  |  |
| Female genital organs | - |  | 2,338 (9.8%) |  |  |
| Urinary tract | - |  | 1,426 (6.0%) |  |  |
| Respiratory/intrathoracic | - |  | 1,223 (5.1%) |  |  |
| Lip, oral cavity and pharynx | - |  | 635 (2.7%) |  |  |
| Thyroid, other endocrine | - |  | 436 (1.8%) |  |  |
| Mesothelial and soft tissue | - |  | 345 (1.4%) |  |  |
| Eye, brain, central nervous system | - |  | 317 (1.3%) |  |  |
| Secondary, unspecified sites | - |  | 277 (1.2%) |  |  |
| Bone and articular cartilage | - |  | 56 (0.2%) |  |  |

*IPAQ: International Physical Activity Questionnaire.*

Supplementary Table 2: Comparison of participants with CMR data.

|  | Control (n=1,538) |  | Cancer history (n=1,051) |  | p-value |
| --- | --- | --- | --- | --- | --- |
| Age, years | 59.1 ± 6.8 |  | 59.0 ± 6.8 |  | 0.50 |
| Female (%) | 892 (58.0) |  | 640 (60.9) |  | 0.15 |
| CMR measurements |  |  |  |  |  |
| Body surface area (m2) | 1.84 ± 0.20 |  | 1.83 ± 0.20 |  | 0.39 |
| LV ejection fraction (%) | 60.1 ± 6.4 |  | 59.2 ± 6.5 |  | **<0.001** |
| LV cardiac output (L/min) | 5.3 ± 1.2 |  | 5.3 ± 1.2 |  | 0.54 |
| LV stroke volume (mL) | 84.9 ± 18.1 |  | 84.1 ± 18.1 |  | 0.30 |
| LV global circumferential strain (%) | -22.6 ± 3.5 |  | -22.2 ± 3.5 |  | **0.001** |
| LV global longitudinal strain (%) | -18.6 ± 2.8 |  | -18.6 ± 2.9 |  | 0.87 |
| LV global radial strain (%) | 46.2 ± 8.5 |  | 45.3 ± 8.8 |  | **0.007** |
| LV end systolic volume (mL) | 57.4 ± 18.6 |  | 58.9 ± 18.9 |  | **0.035** |
| LV end diastolic volume (mL) | 142.2 ± 31.8 |  | 143.0 ± 31.6 |  | 0.51 |
| LV mean myocardial wall thickness (mm) | 5.7 ± 0.8 |  | 5.6 ± 0.7 |  | 0.20 |
| LV myocardial mass (g) | 83.1 ± 20.7 |  | 82.6 ± 20.5 |  | 0.54 |
| LVMi (g/m^2^) | 44.9 ± 8.2 |  | 44.8 ± 8.2 |  | 0.78 |
| LA ejection fraction (%) | 60.3 ± 10.3 |  | 60.4 ± 9.3 |  | 0.84 |
| LA minimum volume (mL) | 29.8 ± 16.8 |  | 29.3 ± 16.4 |  | 0.50 |
| LA maximum volume (mL) | 71.6 ± 23.9 |  | 71.0 ± 23.2 |  | 0.55 |
| minLAVi (mL/m^2^) | 16.1 ± 8.7 |  | 16.0 ± 8.4 |  | 0.63 |
| maxLAVi (mL/m^2^) | 38.9 ± 12.1 |  | 38.8 ± 11.7 |  | 0.81 |
| Categorical output |  |  |  |  |  |
| Abnormal GLS (≥ -16%) (%) | 259 (16.8) |  | 179 (17.0) |  | 0.94 |
| High LAVi (>34 mL/m^2^) (%) | 47 (3.1) |  | 25 (2.3) |  | 0.36 |
| Low LVEF (≤ 55%) (%) | 290 (18.9) |  | 254 (24.2) |  | **0.001** |

*CMR: cardiac magnetic resonance imaging; LA: left atrium; LV: left ventricle; LVMi: left ventricular mass index; LAVi: left atrial volume index; GLS: global longitudinal index; LVEF: left ventricular ejection fraction*

Supplementary Table 3: Comparison of baseline characteristics between participants with normal and low LVEF.

|  | Normal LVEF (n = 2,045) |  | Low LVEF (≤55%) (n = 544) |  | p-value |
| --- | --- | --- | --- | --- | --- |
| Age, years | 58.9 ± 6.8 |  | 59.5 ± 6.9 |  | 0.067 |
| Female (%) | 1,329 (60.6) |  | 203 (37.3) |  | **<0.001** |
| Cancer type (%) |  |  |  |  |  |
| Breast cancer (%) | 375 (18.3) |  | 91 (16.7) |  | 0.42 |
| Haematological malignancies (%) | 84 (4.1) |  | 36 (6.6) |  | **0.018** |
| Other cancer (%) | 383 (18.7) |  | 134 (24.6) |  | **0.003** |
| Years since cancer diagnosis, years | 5.0 ± 8.9 |  | 4.9 ± 7.8 |  | 0.83 |
| Systolic blood pressure, mmHg | 139 ± 19 |  | 139 ± 20 |  | 0.72 |
| Body mass index, kg/m2 | 26.7 ± 4.3 |  | 26.6 ± 3.9 |  | 0.32 |
| Diabetes (%) | 136 (6.7) |  | 39 (7.2) |  | 0.74 |
| Ischemic heart disease (%) | 179 (8.8) |  | 73 (13.4) |  | **0.001** |
| Chronic renal failure (%) | 71 (3.5) |  | 27 (5.0) |  | 0.14 |
| Blood pressure medication (%) | 197 (9.6) |  | 81 (14.9) |  | **0.001** |
| Cholesterol lowering medication (%) | 169 (8.3) |  | 94 (17.3) |  | **<0.001** |
| IPAQ (%) |  |  |  |  | 0.46 |
| Low | 310 (15.2) |  | 93 (17.1) |  |  |
| Moderate | 721 (35.3) |  | 186 (34.2) |  |  |
| High | 648 (31.7) |  | 189 (34.7) |  |  |

*LVEF: Left ventricle ejection fraction; Breast cancer: malignant neoplasms of breast; Blood-related cancer: malignant neoplasms, stated or presumed to be primary, of lymphoid, haematopoietic and related tissue; IPAQ: International Physical Activity Questionnaire*

Supplementary Table 4: Independent associations of LV systolic dysfunction (LVEF<50%).

|  | Clinical variables | |  | Cancer variables | |  | All variables | |
| --- | --- | --- | --- | --- | --- | --- | --- | --- |
|  | PR (95% CI) | p-value |  | PR (95% CI) | p-value |  | PR (95% CI) | p-value |
| Age at consent | 1.03 (1.01-1.04) | **<0.001** |  | 1.03 (1.01-1.04) | **<0.001** |  | 1.02 (1.01-1.04) | **0.004** |
| Female | 0.36 (0.29-0.45) | **<0.001** |  | 0.28 (0.21-0.37) | **<0.001** |  | 0.31 (0.23-0.40) | **<0.001** |
| Cancer history at baseline | 1.28 (0.97-1.66) | 0.077 |  | - | - |  | - | - |
| Breast cancer | - | - |  | 2.05 (1.43-2.90) | **<0.001** |  | 2.01 (1.41-2.83) | **<0.001** |
| Hematological malignancies | - | - |  | 1.29 (0.89-1.78) | 0.16 |  | 1.28 (0.89-1.77) | 0.17 |
| Other cancer | - | - |  | 0.95 (0.67-1.33) | 0.78 |  | 0.94 (0.66-1.31) | 0.74 |
| Years since cancer diagnosis | 1.01 (1.00-1.02) | 0.32 |  | 1.00 (0.99-1.01) | 0.96 |  | 1.00 (0.99-1.01) | 0.99 |
| Blood pressure medication | 1.10 (0.87-1.37) | 0.44 |  | 1.10 (0.87-1.38) | 0.42 |  | 0.82 (0.63-1.05) | 0.12 |
| Diabetes | - | - |  | - | - |  | 1.22 (0.91-1.60) | 0.16 |
| Cholesterol lowering medication | - | - |  | - | - |  | 1.39 (1.08-1.77) | **0.009** |
| Ischemic heart disease | - | - |  | - | - |  | 1.80 (1.44-2.22) | **<0.001** |

*LVEF: left ventricle ejection fraction; OR: odds ratio; CI: confidence interval*

Supplementary Table 5: Multivariable linear regression analyses on key CMR measurements

|  | LV ejection fraction | |  | LV myocardial mass | |  | GLS | |  | GCS | |
| --- | --- | --- | --- | --- | --- | --- | --- | --- | --- | --- | --- |
|  | β (SE) | p-value |  | β (SE) | p-value |  | β (SE) | p-value |  | β (SE) | p-value |
| Age at consent | 0.04 (0.01) | **<0.001** |  | -0.3 (0.03) | **<0.001** |  | -0.003 (0.005) | 0.52 |  | -0.02 (0.01) | **0.001** |
| Female | 3.6 (0.2) | **<0.001** |  | -30.5 (0.4) | **<0.001** |  | -1.4 (0.1) | **<0.001** |  | -2.3 (0.1) | **<0.001** |
| Cancer history at baseline | -0.5 (0.2) | **0.025** |  | -0.2 (0.5) | 0.74 |  | 0.03 (0.1) | 0.80 |  | 0.2 (0.1) | **0.045** |
| Years since cancer diagnosis | 0.003 (0.01) | 0.74 |  | 0.02 (0.02) | 0.38 |  | 0.01 (0.004) | 0.18 |  | -0.004 (0.005) | 0.43 |
| Blood pressure medication | 0.3 (0.2) | 0.31 |  | 5.1 (0.6) | **<0.001** |  | -0.1 (0.1) | 0.50 |  | -0.01 (0.1) | 0.94 |

*LV: left ventricle; GLS: global longitudinal strain; GCS: global circumferential strain; SE: standard error.*

Supplementary Table 6: Multivariable binomial regression analyses on categorical outcomes.

|  | Abnormal GLS (≥ -16%) | |  | High LAVi (>34mL/m^2^) | |
| --- | --- | --- | --- | --- | --- |
|  | PR (95% CI) | p-value |  | PR (95% CI) | p-value |
| Age at consent | 1.00 (0.99-1.01) | 0.77 |  | 1.11 (1.08-1.14) | **<0.001** |
| Female | 0.44 (0.39-0.49) | **<0.001** |  | 0.72 (0.52-0.98) | **0.039** |
| Cancer history at baseline | 1.03 (0.88-1.20) | 0.73 |  | 0.84 (0.53-1.27) | 0.42 |
| Years since cancer diagnosis | 1.00 (0.99-1.00) | 0.53 |  | 1.01 (0.99-1.02) | 0.57 |
| Blood pressure medication | 0.97 (0.84-1.11) | 0.63 |  | 1.60 (1.14-2.21) | **0.005** |

*GLS: global longitudinal strain; LAVi: left atrial volume index; OR: odds ratio; CI: confidence interval.*

Supplementary Figure 1: Histogram of residuals to assess normality


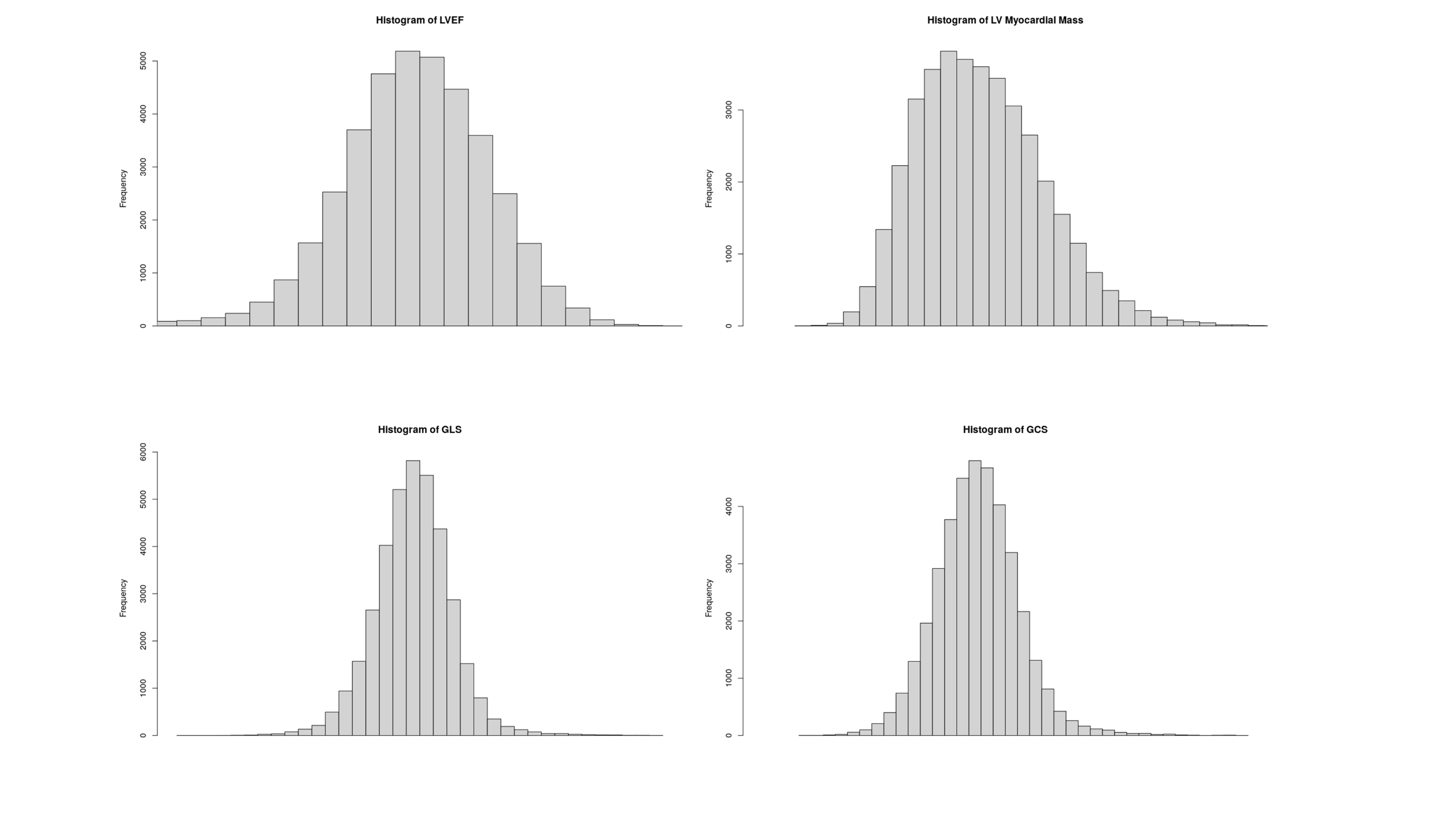


Supplementary Figure 2: Histogram of residuals from linear regression models


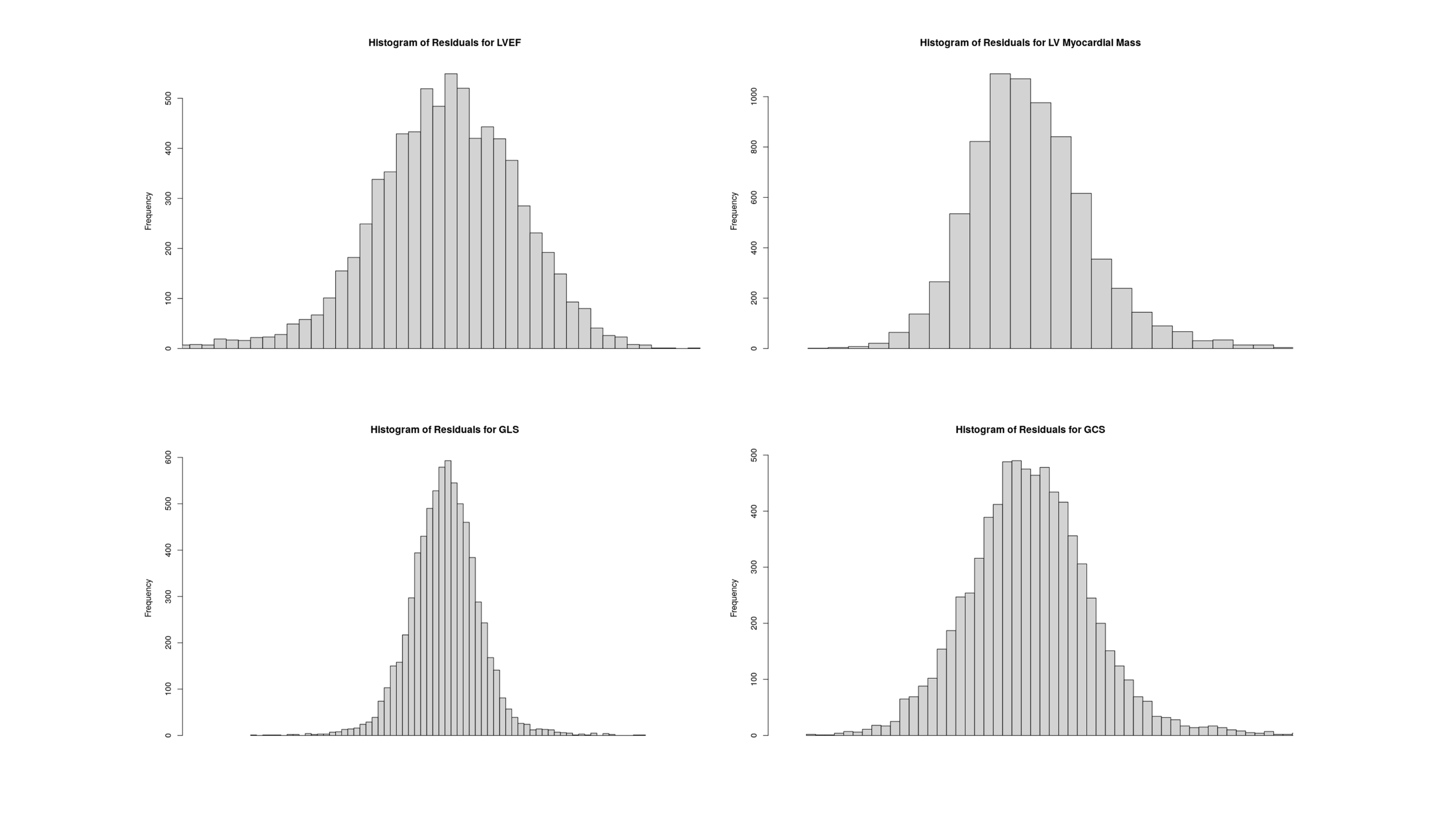

Supplement: online supplemental file 2 [file heartjnl-111-24-s002.docx]
